# Evaluation of GeneXpert and advanced biological laboratories UltraGene HCV diagnostic detection and performance against Roche real time PCR in Myanmar

**DOI:** 10.1016/j.jcv.2024.105653

**Published:** 2024-02-12

**Authors:** Pedro Pisa, Constance Wose Kinge, Charles Chasela, Eula Mothibi, Yin Min Thaung, Hnin T. Thwin, Nay M. Aung, Kara W. Chew, Malini M. Gandhi, Cavenaugh Clint, Thomas Minior, Aye A. Lwin, Morgan J. Freiman, Khin P. Kyi, Yi Y. Sein, Fadzai Marange, Charles van der Horst, Sofiane Mohamed, Matthieu Barralon, Ian Sanne

**Affiliations:** a Department of Strategic Information, Right to Care, Centurion, South Africa; b Department of Human Nutrition, University of Pretoria, Faculty of Health Sciences, Pretoria, South Africa; c Department of Implementation Science, Right to Care, Centurion, South Africa; d Department of Epidemiology and Biostatistics, School of Public Health, Faculty of Health Sciences, University of the Witwatersrand, Johannesburg, South Africa; e Community Partners International, Yangon, Myanmar; f David Geffen School of Medicine at UCLA, Los Angeles, CA, USA; g U.S. Agency for International Development, USA; h Department of Medical Research, Ministry of Health and Sports, Yangon, Myanmar; i Myanmar Liver Foundation, Yangon, Myanmar; j Right to Care, Centurion, South Africa; k University of North Carolina, Chapel Hill, NC. USA; l Advanced Biological Laboratories (ABL) SA.17, rue des Jardiniers, Luxembourg 1835; m Clinical HIV Research Unit, School of Clinical Medicine, Faculty of Health Sciences, University of the Witwatersrand, Johannesburg, South Africa

**Keywords:** Near to real–time testing, HCV RNA, PCR, GeneXpert, Viral load, Hepatitis c virus, Myanmar

## Abstract

**Background:**

Developing countries experience limited access to HCV laboratory tests for different reasons. Providing near to real–time HCV testing and results especially to at–risk populations including those in rural settings for timely initiation to treatment is key. Within a rural Myanmar setting, we compared HCV diagnostic detection and quantification of the GeneXpert, and Advanced Biological Laboratories UltraGene–HCV assays against the gold standard and reference method Roche real–time HCV in Myanmar.

**Methods:**

Blood samples from 158 high–risk individuals were assessed using three different methods at baseline. Results were checked for normality and log transformed. Log differences and bias between methods were calculated and correlated. Pearson’s correlation coefficient was used to determine the association of HCV viral loads across all methods. The level of agreement with the standard method (Roche real time HCV) was assessed using Bland–Altman analyses.

**Results:**

There was a strong positive correlation coefficient between all three methods with GeneXpert and Roche having the strongest, *r* = 0.96, (*p*<0.001). Compared to Roche, ABL (mean difference, 95 % limits of agreement; −0.063 and −1.4 to 1.3 Log10IU/mL) and GeneXpert (mean difference, 95 % limits of agreement; −0.28 and −0.7 to 1.8 Log10IU/mL) showed a good level of agreement with the GeneXpert being slightly superior.

**Conclusion:**

We demonstrate the excellent performance and no-inferiority, in terms of levels of agreements of both GeneXpert and ABL compared to the Roche platform and supporting the use of the POC assays as alternative a cost-effective methods in HCV detection and diagnosis in developing and low resource settings countries.

## 1. Introduction

HCV remains a leading cause of mortality worldwide, causing more than 290,000 deaths in 2019 [1,2]. Though curable Hepatitis C Virus (HCV) infection is associated with high morbidity and mortality and disproportionately affects people in low- and middle-income countries (LMICs) [1,3,4]. Over the years, HCV infection has remained high in certain populations even in low prevalence settings, specifically people who inject drugs (PWID) and HIV-infected men who have sex with men (MSM) [5]. Chronic HCV infection is expected in 55 % to 85 % of untreated cases and is associated with liver cirrhosis, liver failure, hepatocellular carcinoma, and death [4,6].

Effective direct-acting antiviral (DAA) treatment is often available, which can cure HCV infection with 8–12 weeks of therapy, however a significant gap exists in timely diagnosis. with less than 10 % tested and diagnosed in LMICs [1]. In Myanmar the prevalence of HCV and hepatitis B virus (HBV) infection is estimated to be 2.7 % and 6.5 %, respectively [7]. The prevalence of HCV antibody positivity among PWID is estimated at 48.1 %, with treatment access in the region under 1 % [8]. HCV infection is estimated to account for 25 % of hepatocellular carcinoma [5]. The HIV epidemic is concentrated among MSM, PWID, and female sex workers (SW) and ranks among the most serious in Asia [9-11]. HCV/HIV co-infection rates are estimated to between 5 % to 22.8 % with high risk among PWID [10,12-14].

Increased screening and access to treatment for HCV provides an opportunity for integrated screening, linkage, and follow up of HIV/HCV co-infected individuals and management of both HIV and HCV infections. Access to HCV testing in most low- and middle-income countries is costly and limited. A need for reliable non-conventional methods, including Point of Care (POC) approaches especially to at-risk populations including rural settings in Myanmar, for timely initiation to treatment. This paper compares HCV diagnostic detection and quantification of the GeneXpert (Cepheid, CA, USA), and Advanced Biological Laboratories (ABL SA., Luxembourg) Ultra Gene-HCV assays against Roche real–time HCV as the standard in Myanmar.

## 2. Materials and methods

### 2.1. Study sites and population

Enrolment into the main HCV/HIV demonstration project was conducted between December 2017 and December 2018. The participants were recruited at three clinical facilities in Myanmar (the Than Sitt Charity Clinic in Yangon and the Than Sitt Charity Clinic in Mandalay, both operated by the Myanmar Liver Foundation (MLF), and the Waimaw AHRN Clinic in Kachin. Patients were referred to the study sites by civil society groups, the General Practitioners Society, National AIDS Program, Myanmar Anti-Narcotic Association (MANA), Médecins Sans Frontier Holland (MSFH), and Médecins du Monde (MdM). The target populations for the project were people who inject drugs (PWID), men who have sex with men (MSM), and Female Sex Workers (FSW), although initial enrolment included populations outside of these, most of whom could not afford private treatment.

### 2.2. Selection criteria

Eligible participants were HCV viremic, HCV treatment naïve or experienced (prior pegylated interferon [PegIFN] and RBV only), and 18 years or older, with or without HIV-1 co-infection. All participants provided written informed consent.

### 2.3. Measurement of HCV viral load

HCV viral load testing was undertaken for all participants with positive HCV antibody results, using Cepheid GeneXpert, Roche, and Advanced Biological Laboratories (ABL) UltraGene-HCV assays [15]. Plasma samples from HCV-infected subjects were used to isolate HCV RNA. Roche HCV viral load served as the standard of care viral load assay to the validation of GeneXpert HCV viral load. The limits of quantification were 10 IU/mL for GeneXpert, 15 IU/mL for Roche, and 20 IU/mL for ABL.

### 2.4. Xpert^®^ (Cepheid) HCV RNA viral load testing

The Xpert HCV Viral Load (Cepheid, CA, USA), which utilises automated reverse transcriptase polymerase chain reaction (RT-PCR) using fluorescence to detect and quantify RNA, was performed at Ni-Ni Laboratory in Yangon and Kachin following the manufacturer’s protocols [15]. Briefly, approximately 3 mL of whole blood collected in EDTA tubes was centrifuged at 3500 rpm for 15 min to separate the plasma and red blood cells and stored at −20 °C prior to testing. For testing, an aliquot of 1 mL of plasma was loaded unto Xpert cartridges and run for 105 min. The HCV VL Assay quantified all HCV genotypes over the range of 10 to 100,000,000 IU/mL. The results were interpreted automatically by the GeneXpert Instrument System from measured fluorescent signals and embedded calculation algorithms.

### 2.5. ABL (SA) ultragene HCV viral load assays

The ABL HCV viral load testing was performed at the Ministry of Health Department of Medical Research, Yangon, Lower Myanmar. Total RNA was extracted from 140 –L of plasma for all samples using the manufacturer’s instructions for the QIAamp Viral RNA Mini Kit (QIA-GEN). RNA was eluted in 60 μL using the elution buffer. A volume of 8.25 μL of extracted RNA was used as template for RT-qPCR with NS5B and 5′UTR specific primers in a QuantStudio3 System (Thermo Fisher Scientific). Sequences of these primers can be made available upon request. Viral Load Assays are fast and ultra-sensitive real–time PCR tests that provide qualitative detection and quantification of HCV RNA from plasma, serum, whole blood, or dried blood spot samples. Additionally, they generate PCR amplicons of the 5 ’UTR and NS5B genes and which can be used as a reflex testing on positive samples for subtyping through Sanger or Next generation sequencing. The entire protocol described here, encompassing all stages from extraction to RT-qPCR data analysis, required approximately three hours.

### 2.6. COBAS^®^ taqman ^®^HCV quantitative) assay for HCV RNA viral load

The COBAS AmpliPrep/COBAS Taqman HCV Test is an in vitro nucleic acid amplification test for the quantitation of hepatitis C viral (HCV) RNA in human plasma or serum of HCV- infected individuals using the COBAS AmpliPrep Instrument for automated sample processing and the COBAS Taqman Analyzer for the automated amplification and detection. HCV viral load testing was performed at the Ni-Ni Laboratory in Yangon using plasma samples. Briefly, 3 mL of whole blood collected in EDTA was centrifuged at 3500 rpm for 15 min at room temperature. Approximately 1.5 mL of the resultant plasma was transferred into a sterile polypropylene tubes and stored at −20 °C prior to testing. Upon testing, plasma samples and controls were removed from storage sites and equilibrated to ambient temperature before use. Frozen samples were thawed at room temperature and vortexed for 3–5 s before use. All the reagent cassettes were removed from 2 to 8 °C storage and immediately loaded onto the COBAS AmpliPrep Instrument and allowed to equilibrate to ambient temperature on the instrument for at least 30 min before the first specimen was processed. Reagent racks were loaded according to the instructions in the Package Insert. Aliquots of 1000 uL of each specimen and control were transferred to the appropriate bar-coded labeled Input S-tube using a micropipettor with positive displacement RNase-free tips and run according to manufacturer’s instructions (Roche Diagnostics). The COBAS Taqman 48 Analyzer automatically determined the HCV RNA concentration for the specimens and controls. The HCV RNA concentrations were expressed in International Units (IU)/mL.

### 2.7. Statistical analysis

Continuous and categorical variables were summarised as mean (s. d.) and n (%), respectively. Pearson’s correlation coefficient analyses were used to determine the association of HCV viral loads between all methods. The level of agreement with the standard method was assessed using Bland–Altman analyses. Sensitivity and specificity analysis were additionally computed. Data were analysed using STATA 13.0 (Stata-Corp, College Station, TX, USA), and p values tailed at 0.05 were considered statistically significant.

### 2.8. Ethics

The study was conducted in accordance with the Declaration of Helsinki and approved Myanmar Ministry of Health and Sports Institutional Technical and Ethical Review Board, University of Public Health (ITERB-2017/Research/18), the University of the Witwatersrand Human Research Ethics Committee (M17078) and the UCLA Medical Institutional Review Board (#18-00,003). The Boston University Institutional Review Board approved analysis of a de-identified analytic dataset (H-37,820). Informed consent was obtained from all subjects involved in the study.

## 3. Results

**Fig. 1.**
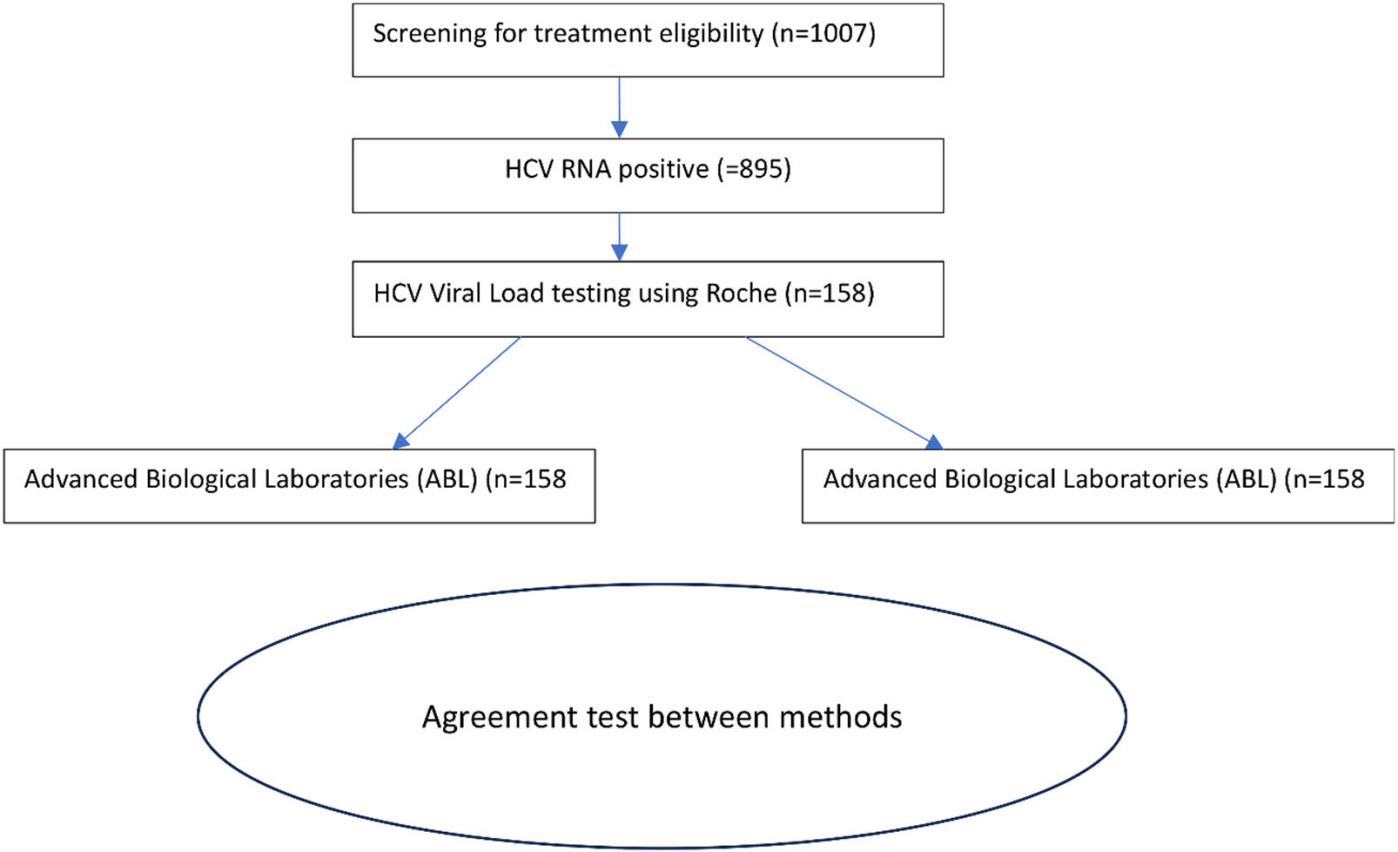
Recruitment of participants in comparing HCV diagnostic detection and quantification.

Fig. 1 presents the study enrolment flow diagram for comparing HCV diagnostic detection and quantification.

**Fig. 2.**
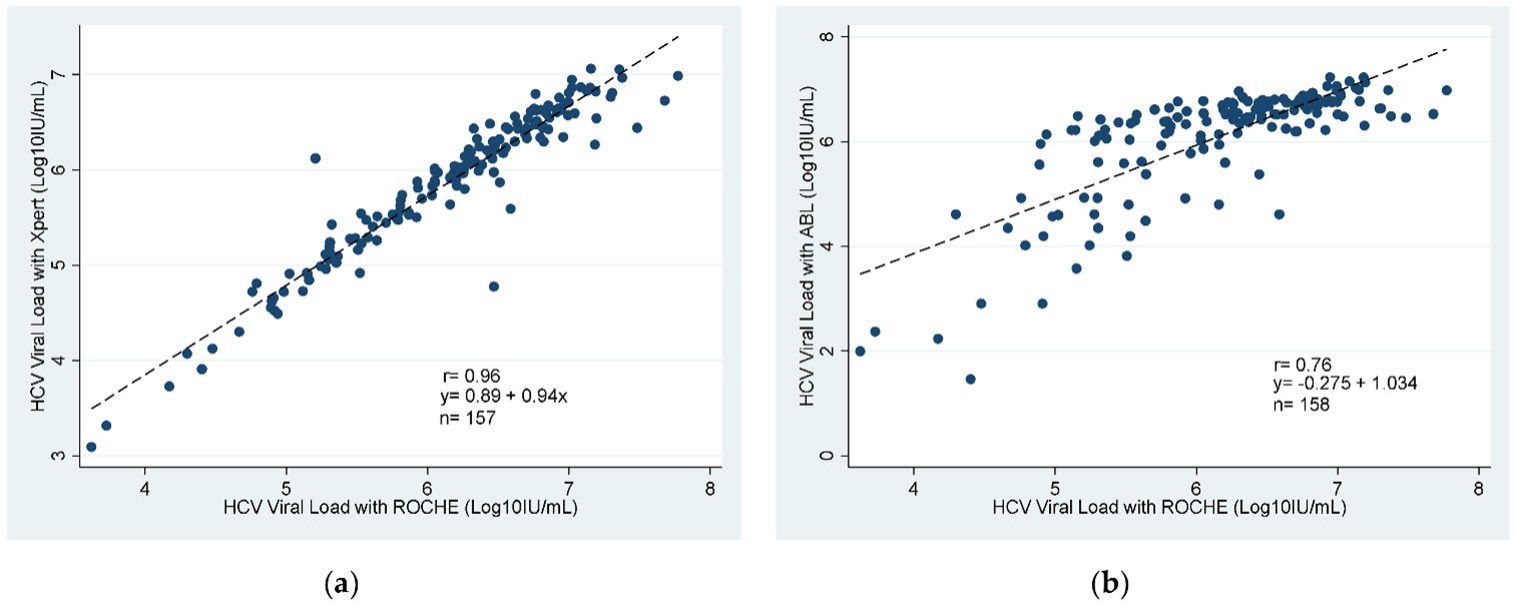
Scatter plots and Pearson correlations for HCV viral loads across between methods: (**a**) Correlation between GeneXpert (Cepheid, CA, USA) and Roche real–time HCV (*n* = 158); (**b**) Correlation between Advanced Biological Laboratories (ABL) UltraGene-HCV assays and Roche real–time HCV (*n* = 158).

Scatter plots of GeneXpert, Roche real–time HCV and Advanced Biological Laboratories (ABL) UltraGene-HCV assays estimates for HCV viral load showed a positive linear relationship between all 3 methods (Fig. 2). The corresponding Pearson’s correlation coefficients indicated significant correlations between all 3 methods measurements of HCV viral load (*p*<0.001), with all correlation coefficients being higher than 0.7. The strongest correlation of 0.96 was observed between GeneXpert and Roche.

**Fig. 3.**
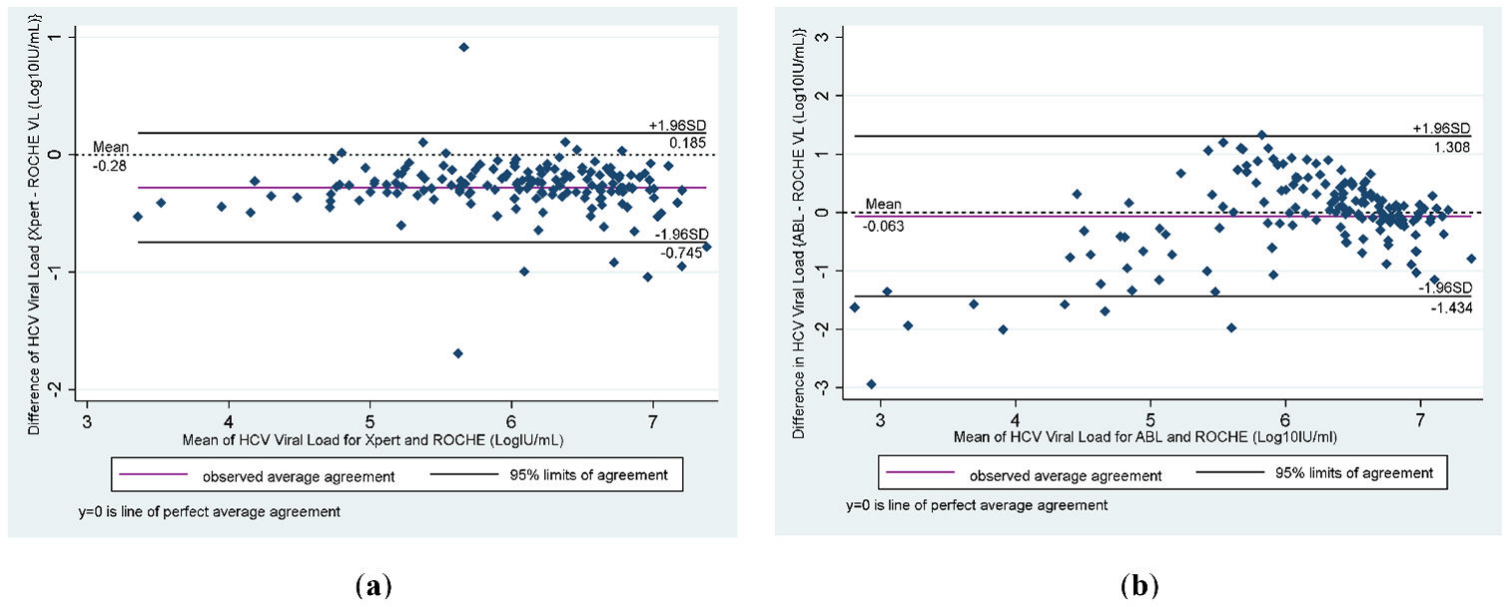
Bland-Altman Analyses in comparison with Roche (standard) for HCV viral loads: (**a**) Bland Altman bias plots between Xpert and Roche; (**b**) Bland Altman bias plots of differences between ABL and Roche.

Bland–Altman analyses of HCV viral load by GeneXpert, Roche real–time HCV and Advanced Biological Laboratories (ABL) UltraGene-HCV assays are presented in Fig. 3. Compared to Roche real–time HCV as the standard, both methods showed a good level of agreement: GeneXpert (Cepheid, CA, USA) (mean difference, 95 % limits of agreement; −0.28 and −0.7 to 0.2 IU/mL) (Fig. 3a) and ABL (mean difference, 95 % limits of agreement; −0.063 and −1.4 to 1.3 Log10IU/mL) (Fig. 3b).

## 4. Discussion

This study aimed to determine whether GeneXpert, and ABL provided comparable measurements of HCV diagnostic detection and quantification against the Roche real–time HCV which is the gold standard and reference method in Myanmar. We found that Pearson’s correlation coefficients indicated significant correlations between all 3 methods measurements of HCV viral load with all correlation coefficients being high. Compared to Roche real–time HCV as the standard, both methods showed a good level of agreement with the GeneXpert method illustrating as a superior alternative to ABL. To our knowledge, this is the first study comparing these innovative POC methods, with the gold standard in at risk populations in Myanmar.

Although all methods seem to provide reliable estimates for HCV diagnostic detection and quantification, accessibility of the gold standard approach in LMICs is limited and often expensive. Myanmar among other comparable LMICs, need reliable and validated non-conventional methods, including POCs approaches defined as medical diagnostic testing at or near the POC that is at the time and place of care. From the current study results, the GeneXpert method, offers a sustainable option for population at risk as it is a small sized instrument system with minimal requirement of infrastructure and can be performed at or near patient care by nonlaboratory- trained individuals such as physicians, nurses, and nursing assistants. The Nucleic acid extraction, amplification, detection, and quantitative of HCV RNA target sequences processes are automated in a simple one-step process following sample collection and preparation as opposed to the multiple step processes employed by the COBAS system, which requires PCR room settings for the large sized COBAS AmpliPrep / COBAS Taqman HCV test instruments in a standard laboratory. More so, the GeneXpert turnaround time for the test is 105 min as opposed to approximately 4 h by the COBAS System [16-18]. Scale up, and increased coverage of the GeneXpert method in low resourced setting in Myanmar, can go a lng way to improve screening levels and access to treatment for HCV providing an opportunity for integrated screening, linkage, and follow up of HIV/HCV co-infected individuals and management of both HIV and HCV infections.

In the current study, we found both methods showed a small mean difference with ABL (mean difference, −0.063) and GeneXpert (mean difference, −0.28) indicating the absence of systematic bias. However, in terms of level of agreements (LOA) for GeneXpert set at −0.7 to 1.8 Log10 IU/mL while ABL was −1.4 to 1.3 IU/mL). Our findings for both methods are comparable to previous studies by Gupta et al. [19] (mean difference of 0.04 log10 IU/mL and LOA of −0.42 and 0.49), McHugh et al. [20] (mean difference of 0.03 log10 IU/mL and LOA of −0.41 to 0.47), and Grebely et al. [21] (mean difference of −0.036 log10 IU/mL and LOA of −0.28 to 0.35).

Limitation is that we did not include the quantification of VL during or at the end of treatment for all samples (only pretreatment samples are reflected in the current analysis). These samples may have low levels of quantifiable HCV RNA [22]. Although our findings suggest that both methods are extremely comparable to the Roche methods, cost evaluations for the two methods were not explored in the current analysis.

A major strength of this study is that implementation was done in a resource-limited clinical setting and high-risk populations for HCV, with locally hired and trained laboratory technicians performing the procedures. Hence, it captures the Myanmar reality on the possible POC options and methods that are possible in the country for HCV detection. Xpert allows VL results to be available at the point of treatment within the same day compared to when reliant on only a central laboratory. While Xpert would be reliable, the number of samples processed per day would be limited if same machine is being used for different diseases and the POC testing depends on one GeneXpert machine. Data utilization including of missing results and retesting can then be done immediately if testing is conducted near POC. Based on the current Myanmar clinical setups, this then facilitates referral into care on the same day and patients no longer need to wait and return for another visit to a health facility to receive their test results. These represent significant advantages for patients, as loss to follow-up tends to increase with longer TATs [2]. Same day diagnosis and treatment has been shown to have significant impact on patient retention, especially for patients living in decentralized and remote areas.

Though the current findings strongly suggest a need to implement POC methods at large scale over conventional platforms, the Xpert platform still has major reported operational and cost constraints. The cost per unit has been reported 17,000 US dollars [23], and Iwamoto et al. [16] reports an entire lab set at about 56 000 US dollars for 2 Xpert platforms. More importantly, the Xpert HCV cartridges contain guanidinium thiocyanate to facilitate the extraction of RNAs but for their disposal they require combustion in high-temperature incinerators which is expensive and need infrastructure [1]. Currently, a new Xpert platform, for use on finger-stick capillary whole blood, showed good performance in short period, compared to the standard Xpert HCV but the platform requires that testing immediately follows sample collection, nevertheless necessitating a similar laboratory set-up [24].

## 5. Conclusion

Our study findings demonstrate the excellent performance and no-inferiority, levels of agreements of both GeneXpert and ABL methods compared to the Roche platform and support the use of the POC assays as a cost-effective alternative, in HCV detection and diagnosis in developing and low resources settings countries.
